# Hypertriglyceridemia in chronic kidney disease: pathophysiological mechanisms, cardiovascular risk, and emerging therapeutics

**DOI:** 10.1186/s12944-026-02862-0

**Published:** 2026-01-20

**Authors:** Dandan Li, Zhanju Liu, Hongwei Jiang

**Affiliations:** 1 Department of Endocrinology, The First Affiliated Hospital, and College of Clinical Medicine of Henan University of Science and Technology, Luoyang, China; 2 Department of Gastroenterology, Shanghai Tenth People’s Hospital, Tongji University School of Medicine, 301 Yanchang Road, Shanghai, China; 3 Luoyang Key Laboratory of Clinical Multiomics and Translational Medicine, Key Laboratory of Hereditary Rare Diseases of Health Commission of Henan Province, Henan Key Laboratory of Rare Diseases, Endocrinology and Metabolism Center, The First Affiliated Hospital, and College of Clinical Medicine of Henan University of Science and Technology, 636 Guanlin Road, Luoyang, China

**Keywords:** Hypertriglyceridemia, Chronic kidney disease, Cardiovascular diseases, Lipoprotein lipase, Inflammation, Fibrosis, PPAR alpha, RNAi therapeutics

## Abstract

Hypertriglyceridemia (HTG) is highly prevalent among patients with chronic kidney disease (CKD) and plays a critical role in both the progression of nephropathy and the increased risk of cardiovascular events. The underlying pathophysiology involves metabolic disturbances of triglyceride (TG)-rich lipoproteins, leading to lipid accumulation within renal cells. This accumulation triggers lipotoxicity, oxidative stress, and inflammatory cascades that ultimately contribute to renal fibrosis. In advanced stages of CKD, statins demonstrate limited efficacy, whereas emerging therapeutic agents show promising potential. RNA-based gene therapies targeting apolipoprotein C-III and angiopoietin-like protein 3 effectively reduce TG levels while maintaining a favorable renal safety profile. Additionally, agents such as pemafibrate, PCSK9 inhibitors, sodium–glucose cotransporter-2 inhibitors, and glucagon-like peptide-1 receptor agonists not only lower lipid levels but also confer significant cardiorenal protective effects. Consequently, the management of HTG in CKD should transition from a singular lipid-lowering focus to a comprehensive, stratified approach that targets the interconnected pathways of lipotoxicity, inflammation, and fibrosis, with the ultimate goal of improving cardiorenal outcomes.

## Introduction

The rising prevalence of diabetes, hypertension, and obesity has contributed to a continuous increase in the global burden of chronic kidney disease (CKD). According to the 2024 Global Burden of Disease Study, CKD affects approximately 9.5% of the worldwide population and is projected to become the leading cause of years of life lost by 2040 [1, 2]. Cardiovascular disease (CVD) constitutes a major complication and the primary cause of mortality among CKD patients. Both reduced estimated glomerular filtration rate (eGFR) and elevated urinary albumin-to-creatinine ratio have been independently linked to heightened cardiovascular mortality risk [3, 4]. CKD-associated cardiovascular pathologies, including coronary heart disease (CHD) and heart failure (HF), frequently arise from shared risk factors such as dyslipidemia, hyperglycemia, hypertension, oxidative stress, and inflammation [5].

Among the various dyslipidemias, hypertriglyceridemia (HTG) is especially prevalent in CKD populations and has been strongly implicated in the initiation and progression of renal injury [6]. Importantly, HTG not only promotes lipid accumulation and injury within the kidney but also serves as a crucial metabolic nexus connecting renal dysfunction with systemic cardiovascular risk. Although the cardiovascular consequences of HTG in the general population and lipid management strategies in CKD have been extensively investigated [7, 8], the precise mechanisms through which HTG mediates the bidirectional “renal–cardiac cross-talk and injury” in CKD remain incompletely understood.

This review systematically explores the mechanistic pathways by which HTG perpetuates the “lipotoxicity–inflammation–fibrosis” vicious cycle linking renal and cardiovascular systems. Building on this foundation, we propose an evidence-based, phenotype-driven management framework for addressing HTG in patients with CKD.

## Prevalence of HTG in patients with CKD

Elevated triglyceride (TG) levels and reduced high-density lipoprotein cholesterol (HDL-C) are the most prevalent lipid abnormalities observed in patients with CKD, whereas alterations in low-density lipoprotein cholesterol (LDL-C) are comparatively less pronounced [9–11]. HTG is a frequent and metabolically active complication that occurs across all stages of CKD, influenced by factors including disease etiology, CKD stage, therapeutic interventions, and patient age. As renal function deteriorates, the prevalence of HTG correspondingly increases. Although lipid profiles may appear near normal in early-stage CKD, cross-sectional studies indicate that up to 60% of patients in stages 3 to 5 exhibit TG concentrations exceeding 150 mg/dL [12]. Certain CKD subpopulations experience particularly high rates of HTG; for instance, over 60% of patients with nephrotic syndrome develop HTG due to massive proteinuria disrupting lipoprotein metabolism [12]. Among dialysis recipients, HTG prevalence remains elevated at 45–50%, with peritoneal dialysis patients often presenting with more severe dyslipidemia, partly attributable to glucose-based dialysis solutions and protein losses [12]. Notably, HTG is also common in pediatric CKD populations, affecting approximately one-third to three-quarters of children across different cohorts [13, 14]. Variations in reported prevalence likely reflect differences in study populations, CKD etiologies, and dietary patterns. Collectively, these data underscore HTG as a common, multifactorial metabolic disturbance in CKD, warranting further investigation into its clinical implications.

## Clinical relevance of HTG in CKD

HTG is not only a prevalent laboratory abnormality in CKD but also an independent and modifiable risk factor contributing to the onset and progression of renal dysfunction. Epidemiological evidence consistently demonstrates a graded relationship between TG levels and adverse renal outcomes, as summarized in Table 1.

**Table 1:** Summary of landmark observational studies linking HTG with CKD progression and cardiovascular outcomes

Study Design/Ref.	Population	Sample Size	TG Level/Definition	Primary Endpoint	Key Findings
Retrospective Cohort [15]	General Population	911,360	Elevated (in quantiles)	Progression to advanced CKD (stages 4–5)	Elevated TG was linked to a 28% higher risk of progressing to advanced CKD (aHR 1.28, 95% CI: 1.15–1.43), especially in those with preserved kidney function at baseline.
Retrospective Cohort [16]	General Population	44,964	Per 50 mg/dL increase	Composite renal endpoint (significant eGFR decline or ESRD)	Each 50 mg/dL increase in TG level was associated with a 6.2% higher risk of eGFR decline (OR: 1.062, 95% CI: 1.039–1.086) and a 17.4% higher risk of ESRD (OR: 1.174, 95% CI: 1.070–1.289).
Retrospective Cohort [17]	High-risk patients (DM or ASCVD) on statins	73,388	≥ 150 mg/dL vs. optimal levels	Hospitalization for kidney disease	The elevated-TG cohort (TG ≥ 150 mg/dL) had a 31% higher hospitalization rate for new-onset CKD (HR 1.311, 95% CI: 1.228–1.401), and the high-TG cohort (TG 200–499 mg/dL) had a 45% higher rate (HR 1.451, 95% CI: 1.339–1.572), compared to the reference group (TG < 150 mg/dL and HDL-C > 40 mg/dL).
Prospective Cohort [18]	New-onset T2DM	11,142	≥ 150 mg/dL	Incident DN	High TG at baseline (OR: 1.37, 95% CI: 1.07–1.74) and at follow-up (OR: 1.71, 95% CI: 1.35–2.16) were associated with higher DN risk. Patients with high TG at both baseline and follow-up had a greater risk than those with consistently normal TG (OR: 1.65, 95% CI: 1.23–2.22).

In the general population, HTG exhibits a dose-dependent association with CKD risk. Large-scale cohort studies have confirmed that even moderate elevations in TG levels significantly increase the likelihood of progression to advanced CKD or clinically relevant declines in eGFR [15]. Specifically, each 50 mg/dL increment in TG concentration is associated with a 6% to 17% higher risk of renal endpoint events [16]. Importantly, this association persists among individuals with preserved baseline renal function (eGFR ≥ 60 mL/min/1.73 m²), indicating that HTG contributes to renal impairment early in disease development [15].

Despite statin therapy, persistent HTG remains a significant residual risk factor for renal outcomes [19]. In high-risk populations with diabetes or atherosclerotic CVD (ASCVD), elevated TG levels (≥ 150 mg/dL) correlate with a 30% to 45% increased risk of kidney disease–related hospitalization, underscoring that statins alone do not fully mitigate dyslipidemia-associated renal risk [17]. Prospective data from patients with type 2 diabetes further demonstrate that both baseline and sustained HTG independently predict the development of diabetic kidney disease [18]. Notably, long-term TG control appears to modify this risk; patients achieving normalization of TG levels during follow-up showed no significant increase in kidney disease–related hospitalizations, highlighting the importance of sustained TG management in delaying CKD progression [18].

Collectively, these findings establish HTG not merely as a biomarker but as a pathogenic contributor to renal disease progression.

## CVD risk associated with HTG and CKD

HTG functions not only as a risk factor for CKD progression but also as a critical metabolic nexus linking renal impairment with systemic cardiovascular damage [20, 21]. Within the CKD milieu, HTG acts as a metabolic accelerator that markedly exacerbates the already elevated risk of ASCVD in this population [22].

Robust epidemiological data have established HTG as an independent risk factor for ASCVD in the general population [23–25]. Among CKD patients, this risk is synergistically amplified by coexisting pathophysiological conditions such as insulin resistance, chronic inflammation, and oxidative stress. Together, these factors facilitate the accumulation of atherogenic TG-rich lipoprotein (TRL) remnant particles and potentiate vascular inflammation and endothelial dysfunction [26–28], thereby substantially increasing residual cardiovascular risk. Supporting this, an analysis of 9,270 CKD patients enrolled in the SHARP trial demonstrated that elevated TG levels are independently associated with a higher incidence of atherosclerotic vascular events, with this relationship remaining consistent regardless of renal function, dialysis status, or lipid-lowering therapy [29].

Genetic evidence further supports a causal role for HTG in cardiovascular risk. Mendelian randomization analyses indicate that the association between TRL/remnant cholesterol (TRL/remnant-C) and CHD risk is independent of apolipoprotein B (ApoB) and LDL-C. Notably, the cardiovascular risk per unit increase in TRL/remnant-C exceeds that conferred by LDL-C [30]. These insights underscore the necessity, in CKD patients, of complementing rigorous LDL-C management with targeted and aggressive control of HTG to reduce TRL/remnant-C, thereby addressing a critical component of residual cardiovascular risk.

Given the compelling evidence implicating HTG in both CKD progression and heightened cardiovascular risk, further research is warranted to elucidate the underlying pathophysiological mechanisms by which HTG drives renal injury and accelerates disease development. Such understanding will be instrumental in designing targeted therapeutic strategies aimed at mitigating these risks.

## Mechanism of renal injury caused by HTG

HTG-induced renal injury arises from both systemic and local disruptions in renal lipid metabolism, predominantly driven by lipid accumulation, cytotoxicity, and the ensuing inflammatory and fibrotic responses.

### Lipid metabolism dysregulation and intrarenal lipid accumulation

In the context of CKD, several pathological factors, including uremic toxin accumulation, insulin resistance, and secondary hyperparathyroidism [31, 32], synergistically disrupt the regulation of the lipoprotein lipase (LPL) system. This dysregulation is characterized by elevated levels of potent LPL inhibitors, such as Apolipoprotein C-III (ApoC-III) and angiopoietin-like protein 4 (ANGPTL4) [33, 34], alongside reduced concentrations of LPL activators, including apolipoprotein A-V [35, 36]. The resultant suppression of LPL activity impairs the clearance of circulating TRLs, leading to the accumulation of atherogenic remnant particles [37]. Simultaneously, diminished local lipolytic function in renal resident cells, such as podocytes and proximal tubular epithelial cells, promotes abnormal intracellular lipid accumulation [38–40], marking the earliest phase of renal lipotoxic injury.

### Lipotoxicity in renal cells: oxidative stress and cellular dysfunction

Excessive lipid accumulation, particularly of free fatty acids and TGs, within podocytes and proximal tubular epithelial cells directly induces cellular injury. This damage is mediated through mechanisms including mitochondrial dysfunction, endoplasmic reticulum stress, and increased generation of reactive oxygen species (ROS) [41]. Concurrently, reduced activity of key fatty acid oxidation enzymes such as carnitine palmitoyltransferase 1, along with dysregulation of upstream regulators like adenosine monophosphate-activated protein kinase, impairs fatty acid β-oxidation and decreases ATP production. In podocytes, these disruptions initiate apoptosis and detachment, undermining the integrity of the glomerular filtration barrier and resulting in proteinuria [42, 43]. In proximal tubular epithelial cells, which rely heavily on fatty acid oxidation for energy, these metabolic perturbations lead to brush border degradation and loss of cellular polarity [44–46], accompanied by pronounced activation of tubulointerstitial inflammatory pathways [47].

### Inflammation and fibrosis

Lipotoxicity-induced cellular stress and ROS serve as upstream signaling mediators that activate key inflammatory pathways, notably nuclear factor kappa- B (NF-κB). This activation promotes the production of pro-inflammatory cytokines, including interleukin-6 (IL-6) and tumor necrosis factor-alpha (TNF-α), thereby sustaining chronic inflammation within the kidney [48–50]. Concurrently, the expression of pro-fibrotic mediators such as transforming growth factor-beta (TGF-β) is upregulated, driving the differentiation of renal fibroblasts into myofibroblasts. Additionally, infiltrating macrophages may undergo phenotypic shifts under inflammatory stimuli, further contributing to fibrosis [51, 52]. These activated cells secrete excessive extracellular matrix components while endogenous anti-fibrotic mechanisms, such as relaxin-2, are suppressed, culminating in pathological matrix accumulation and progressive renal fibrosis. This cascade constitutes a central pathological pathway leading to end-stage renal disease [53, 54].

### Therapeutic implications: from mechanism to target conversion

The cascade of lipotoxicity-induced injury presents multiple promising targets for therapeutic intervention. Enhancing LPL activity or inhibiting its key regulators, such as through RNA-based therapies targeting ApoC-III or angiopoietin-like protein 3 (ANGPTL3), can effectively reduce lipid overload at its source. Anti-inflammatory agents, including high-purity icosapent ethyl (IPE), may attenuate lipotoxic injury by mitigating inflammatory responses. Additionally, sodium-glucose cotransporter-2 inhibitors (SGLT2i) and glucagon-like peptide-1 receptor agonists (GLP-1 RAs) have demonstrated renal protective effects by multiple mechanisms, such as alleviating glomerular hypertension and suppressing inflammation and fibrosis. As a result, the management of HTG in CKD is increasingly shifting toward an integrated, mechanism-based therapeutic strategy that targets these interconnected pathological pathways.

## Advances in pharmacotherapy for HTG in CKD

When managing TG levels in patients with CKD, it is crucial to evaluate both the lipid-lowering efficacy and renal safety profiles of therapeutic agents. Table 2 provides a summary of the mechanisms of action, effects on lipid parameters, evidence supporting cardiovascular and renal benefits, and key considerations relevant to CKD for commonly used TG-lowering medications.

**Table 2:** Comparative Pharmacological interventions for HTG in chronic kidney disease

Drug Class	Representative Agents	Core Mechanism of Action	TG Reduction	LDL-C Reduction	CVD/Renal Benefit Evidence	Key Considerations in CKD
First-line Therapies: Primary TG-Lowering Agents						
Fibrates	Fenofibrate, Pemafibrate	Activate PPAR-α, promote fatty acid oxidation [55]	30–50%	5–15% (may ↑)	Pemafibrate shows superior renal safety in pre-clinical and clinical studies [56]	Traditional fibrates require dose adjustment by eGFR; Pemafibrate does not and may improve eGFR [57–61]
High-Purity EPA	Icosapent Ethyl	Multiple (anti-inflammatory, antioxidant), precise target unclear	~ 20–30% [62]	↔	REDUCE-IT: Significant MACE reduction; pronounced benefit in CKD subgroup [63]	No dose adjustment needed. Associated with increased risk of atrial fibrillation and bleeding
ApoC-III Inhibitors	Olezarsen (ASO)	Degrade ApoC-III mRNA, enhance LPL activity [64]	~ 50% [65, 66]	15–20%	Cardiovascular outcome trials ongoing.	GalNAc-conjugated, liver-targeted; favorable renal safety profile in trials to date.
ANGPTL3 Inhibitors	Evinacumab (mAb)	Neutralize ANGPTL3 protein, enhance LPL/EL activity [67]	~ 50% [68]	~ 50%	Approved for HoFH; limited CVD outcome data.	Not renally excreted; expected favorable renal safety.
Ancillary Therapies: Agents with Moderate TG-Lowering Effects						
Statins	Atorvastatin, Rosuvastatin	Inhibit HMG-CoA reductase, upregulate LDL receptor [69]	10–20% [70]	30–50%	Clear benefit in non-dialysis CKD; limited benefit in ESRD (4D, AURORA trials) [71, 72]	Use with caution if eGFR < 30; monitor for myopathy. Not recommended to initiate in dialysis patients
PCSK9 mAbs	Evolocumab, Alirocumab	Inhibit PCSK9, increase available LDL receptors [73]	15–25% [74]	50–60%	FOURIER/ODYSSEY: MACE reduction, consistent benefit in CKD subgroups [75, 76].	No dose adjustment in any stage of CKD; subcutaneous injection.
Bempedoic Acid	Nexletol^®^	Inhibits ATP-citrate lyase (upstream of statins) [77]	10–20% [78]	20–25%	CLEAR Outcomes: MACE reduction in statin-intolerant patients; consistent in CKD [79]	Not renally excreted; no dose adjustment in any CKD stage. Associated with an increased risk of gout.
SGLT2i	Dapagliflozin, Empagliflozin	Promote urinary glucose excretion, improve insulin sensitivity [80]	10–15% [81]	Slight ↑	CREDENCE/DAPA-CKD: Significant slowing of CKD progression and heart failure benefits [82, 83]	Can be initiated if eGFR ≥ 20; an initial transient eGFR drop may occur. Foundational cardiorenal therapy
GLP-1 RAs	Semaglutide, Liraglutide	Delay gastric emptying, suppress appetite, weight loss [84]	10–20% [84]	5–10%	Reduce MACE risk; FLOW trial showed significant renal benefit [85, 86]	No dose adjustment; main side effects are gastrointestinal. Foundational cardiorenal therapy.

*HTG* Hypertriglyceridemia, *TG *Triglycerides, *LDL-C *Low-Density Lipoprotein Cholesterol, *CVD* Cardiovascular Disease, *CKD* Chronic Kidney Disease, *MACE* Major Adverse Cardiovascular Events, *ESRD *End-Stage Renal Disease, *↑* Increase, ↓ Decrease, ↔ No significant change/Mixed effects

### First-line therapies: primary TG–lowering agents

#### Fibrates and the renal–safe profile of pemafibrate

**Fig. 1:**
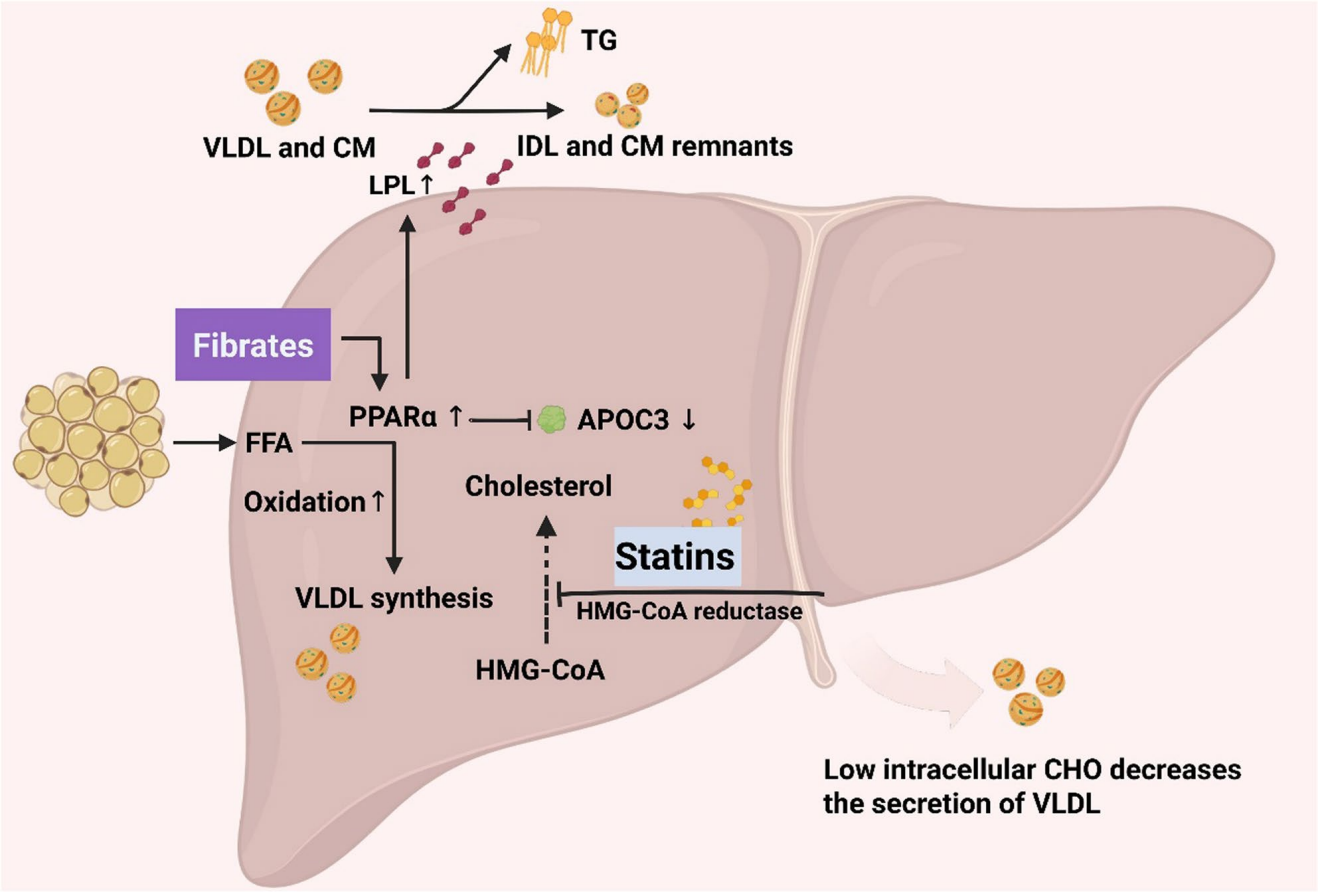
The action sites of traditional TG-regulating drugs. Fibrate drugs activate PPARα, enhancing fatty acid oxidation and reducing TG levels. Statins inhibit HMG-CoA reductase, thereby suppressing cholesterol synthesis and indirectly reducing the secretion of VLDL. PPARα. HMG-CoA, 3-hydroxy-3-methylglutaryl coenzyme A.‌ Source: Created with BioRender.com

Figure 1 illustrates the mechanism by which fibrates lower TG levels. Traditional fibrates, such as fenofibrate, have limitations in patients with CKD due to their potential to increase serum creatinine and reduce eGFR. In contrast, pemafibrate, a novel selective peroxisome proliferator-activated receptor alpha (PPARα) modulator, is not renally metabolized, thereby substantially lowering the risk of renal impairment [55]. Pharmacokinetic studies demonstrate that pemafibrate does not significantly accumulate even in patients with severe renal insufficiency (eGFR < 30 mL/min/1.73 m²) [57], and clinical evidence suggests it may even improve eGFR [58]. These attributes position pemafibrate as a safer and potentially more effective fibrate option for CKD patients with HTG [56, 59, 60].

#### Omega-3 fatty acid

**Fig. 2:**
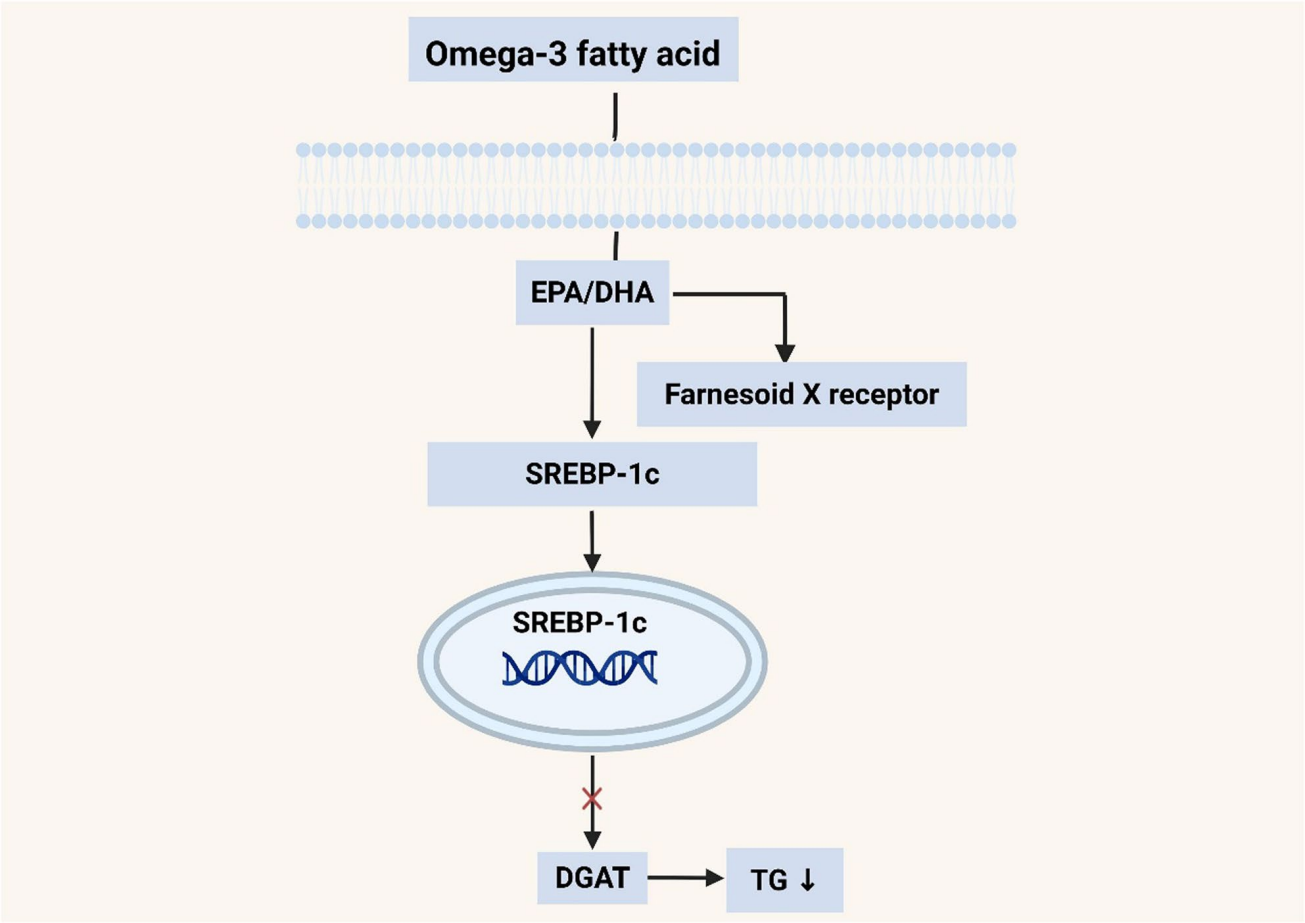
Mechanisms of omega-3 fatty acids reduce hepatic TG synthesis. EPA/DHA activate nuclear receptor FXR to inhibit the activity of transcription factor SREBP-1c and directly inhibit the key synthetic enzyme DGAT in the cytoplasm, jointly reducing TG production. EPA. DHA. FXR, Farnesoid X Receptor. SREBP-1c, Sterol Regulatory Element-binding protein-1c. DGAT, Diacylglycerol Acyltransferase. Solid arrows represent activation or metabolic flux; Red “×” represents inhibition. Source: Created with BioRender.com

High-dose omega-3 fatty acids (2–4 g daily), particularly eicosapentaenoic acid (EPA) and docosahexaenoic acid (DHA), can reduce TG levels by approximately 20–30% [62], with the underlying mechanisms illustrated in Fig. 2. These mechanisms include the inhibition of hepatic TG synthesis, enhanced beta-oxidation of fatty acids, and increased clearance of TRLs.

In cardiovascular outcomes trials, IPE demonstrated significant cardiovascular benefits in high-risk populations, including patients with CKD, as evidenced by the REDUCE-IT study [63]. Conversely, the STRENGTH trial [87] did not observe similar benefits, potentially due to differences in drug formulation and comparator selection (see Table 3). Notably, subgroup analyses from REDUCE-IT indicated that IPE not only provides cardiovascular protection in high-risk patients with comorbid CKD but also exhibits a favorable renal safety profile [63]. Based on this robust evidence, IPE has been approved by the U.S. Food and drug administration (FDA) for cardiovascular risk reduction and is recommended as an adjunctive therapy for high-risk patients on statins in the 2019 European society of cardiology/European atherosclerosis society (ESC/EAS) guidelines [89]. Therefore, for CKD patients with persistent HTG despite statin therapy, IPE represents a valuable treatment option offering TG reduction, cardiovascular protection, and renal safety.

**Table 3:** Key clinical trial outcomes for Lipid-Lowering and cardiorenal therapies in CKD populations

Trial Name (Drug)	Patient Population	Follow-up Duration	Primary Endpoint	Result (Intervention vs. Control)	Impact on eGFR/Renal Events
SHARP (Simvastatin + Ezetimibe) [88]	9,270 patients with CKD (including dialysis and non-dialysis)	4.9 years	Major atherosclerotic events	RR 0.83 (0.74–0.94) Significant reduction	Did not significantly slow eGFR decline, but reduced ESRD risk.
4D (Atorvastatin) [71]	1,255 patients with T2D on HD	4 years	Cardiac death, nonfatal MI, stroke	HR 0.92 (0.77–1.10) Not Significant (NS)	No significant impact.
AURORA (Rosuvastatin) [72]	2,776 patients on HD	3.8 years	CV death, nonfatal MI, stroke	HR 0.96 (0.84–1.11) NS	No significant impact.
REDUCE-IT (Icosapent Ethyl) [63]	High-risk patients, 22% with CKD	4.9 years	MACE	HR 0.75 (0.68–0.83) Significant reduction; CKD subgroup HR 0.71	eGFR remained stable during the trial.
CREDENCE (Canagliflozin) [83]	Patients with T2D and CKD	2.6 years	Renal composite (ESRD, 2x creatinine, renal/CV death)	HR 0.70 (0.59–0.82) Significant reduction	Significantly slowed eGFR decline.
DAPA-CKD (Dapagliflozin) [82]	Patients with CKD with and without T2D	2.4 years	Renal composite (≥ 50% eGFR decline, ESRD, renal/CV death)	HR 0.56 (0.45–0.68) Significant reduction	Significantly slowed eGFR decline.
FLOW (Semaglutide) [85]	Patients with T2D and CKD	3.4 years	Renal composite (ESRD, eGFR decline ≥ 50%, renal/CV death)	HR 0.76 (0.66–0.88) Significant reduction	Significantly slowed eGFR decline.
CLEAR Outcomes (Bempedoic Acid) [79]	Statin-intolerant high-risk patients, 21% with CKD	3.4 years	MACE	HR 0.87 (0.79–0.96) Significant reduction; Consistent in CKD subgroup	Safety profile consistent in CKD vs. non-CKD.
FOURIER (Evolocumab) [75]	ASCVD patients, 4,443 with CKD	2.2 years	MACE	HR 0.85 (0.79–0.92) Significant reduction; Consistent in CKD subgroup	eGFR change similar to placebo.

*CKD* Chronic Kidney Disease, *HD* Hemodialysis, *T2D* Type 2 Diabetes, *MACE* Major Adverse Cardiovascular Events, *MI* Myocardial Infarction, *CV* Cardiovascular, *ESRD *End-Stage Renal Disease, *HR* Hazard Ratio, *RR* Relative Risk, *NS* Not Significant

#### Emerging gene-targeted therapies

Novel RNA-targeted therapies, including acetylgalactosamine (GalNAc)-conjugated small interfering RNA (siRNA) and antisense oligonucleotides (ASO), have demonstrated significant and sustained TG reductions by directly silencing key genes involved in TG metabolism, such as ApoC-III and ANGPTL3. These therapies represent promising future treatment options for severe or refractory HTG.

##### Therapeutics targeting ApoC-III

ApoC-III is a critical regulator of TRL metabolism. Inhibiting ApoC-III enhances the lipolytic processing of TRL particles and promotes their hepatic clearance. Currently, three investigational agents targeting ApoC-III have demonstrated significant efficacy:

##### Volanesorsen

This first-generation ASO lacks GalNAc conjugation. A meta-analysis of three randomized controlled trials (RCTs) involving 207 patients with severe HTG reported that after an average treatment duration of 8.1 months, 84% of volanesorsen-treated patients achieved TG levels below 500 mg/dL, compared to only 35% in the placebo group [90–93].

##### Olezarsen (GalNAc-ASO)

**Fig. 3:**
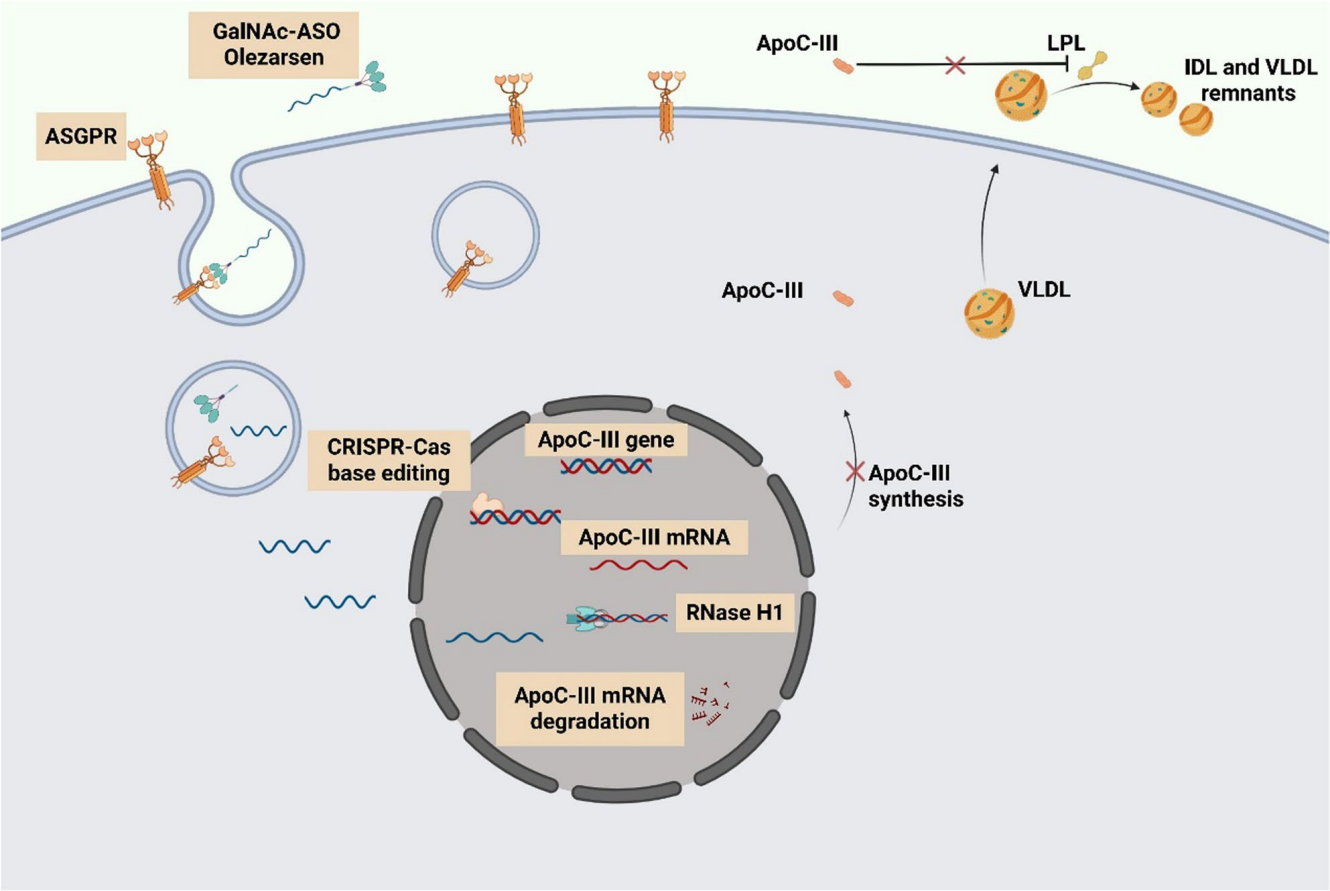
The mechanism of action of gene therapy targeting ApoC-III. This figure illustrates the novel gene therapy targeting ApoC-III, in which ASO bind to ApoC-III mRNA and recruit ribonuclease H1 for its degradation, reducing ApoC-III protein synthesis. Source: Created with BioRender.com

A next-generation hepatocyte-targeted ASO with improved safety and efficacy profiles [64]. The gene therapy approach and underlying mechanism are illustrated in Fig. 3. In a phase IIb trial involving patients with moderate HTG (150–499 mg/dL) and elevated cardiovascular risk or severe HTG (≥ 500 mg/dL), treatment with 50 mg and 80 mg doses of olezarsen resulted in TG reductions of 49.3% and 53.1%, respectively, compared with placebo. The therapy also significantly lowered ApoC-III, ApoB, and non-HDL cholesterol levels. Clinically significant hepatic, renal, or platelet abnormalities were rare and comparable to placebo [65]. Phase III trial results are similarly promising; in patients with genetically confirmed familial chylomicronemia syndrome (FCS), 80 mg of olezarsen reduced TGs by 43.5% at 6 months and decreased ApoC-III levels by 73.7%. Renal safety profiles were comparable between the olezarsen and placebo groups [66].

##### Plozasiran (GalNAc-siRNA)

Plozasiran is a first-in-class subcutaneous RNA interference therapy that silences hepatic ApoC-III mRNA via the GalNAc-siRNA conjugate platform. By enhancing TRL lipolysis and clearance, ploza-siran achieves substantial and sustained reductions in circulating TG levels.

The U.S. FDA has recently approved plozasiran as an adjunct to diet therapy for lowering TG levels in adult patients with FCS, primarily based on the positive outcomes of the global phase III PALISADE trial [94]. This study demonstrated that plozasiran significantly and sustainably reduced TG levels by approximately 80% in patients with FCS and lowered ApoC-III levels by 88% to 94%, while exhibiting a favorable renal safety profile [94].

Beyond FCS, plozasiran is currently being evaluated in broader populations with HTG. The SHASTA-2/3 phase II/III trial is assessing its efficacy in patients with severe HTG, while the MUIR phase II/III study targets individuals with mixed dyslipidemia [95, 96]. These ongoing trials will further establish the efficacy and safety of plozasiran in high-risk cardiovascular populations and clarify its therapeutic role in managing complex lipid disorders.

##### Therapeutics targeting ANGPTL3

**Fig. 4:**
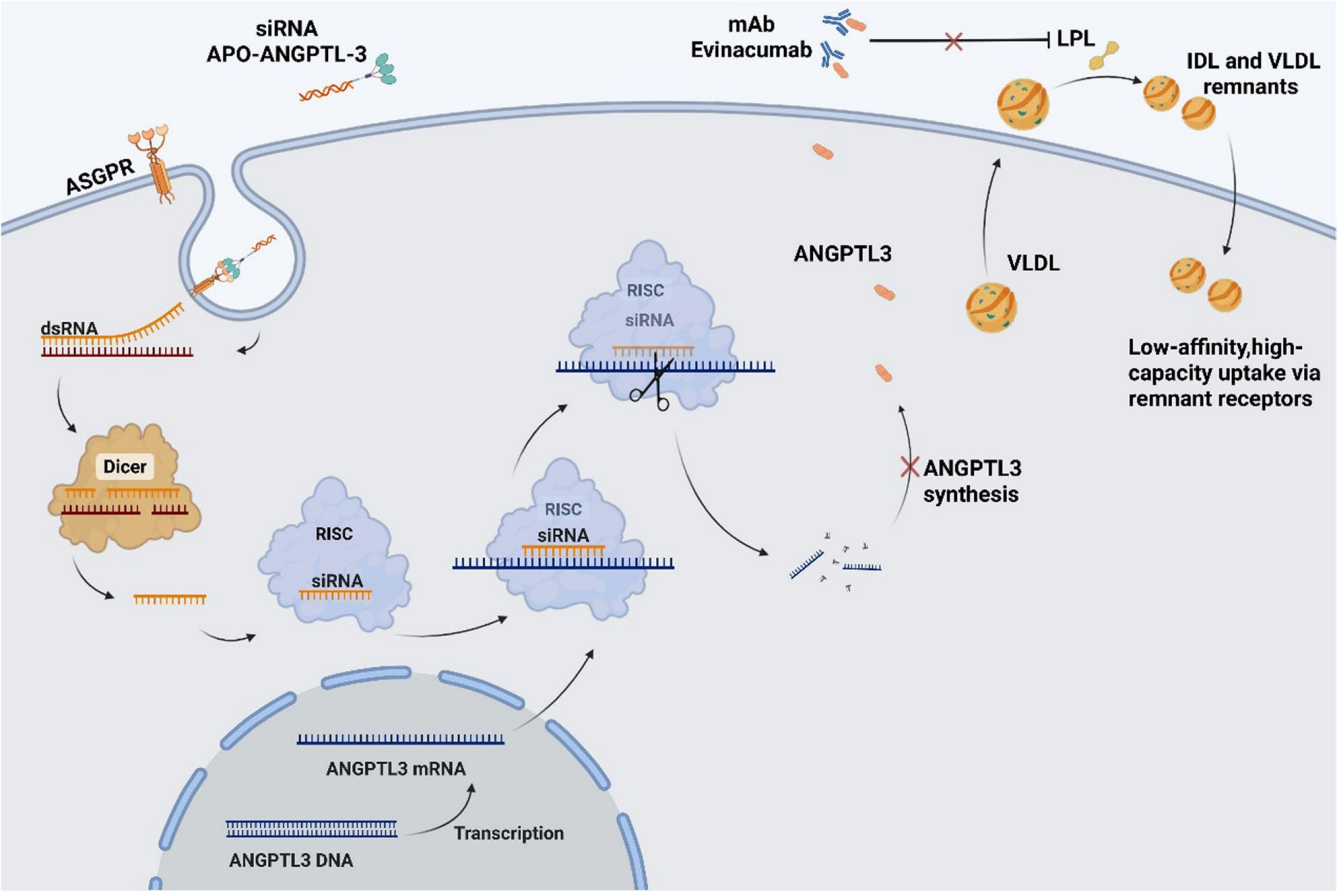
Therapeutic strategies to inhibit ANGPTL3 function. This figure compares two approaches targeting ANGPTL3. (Left) GalNAc-conjugated siRNA against ANGPTL3 is taken up by hepatocytes and silences ANGPTL3 mRNA through the RNA-induced silencing complex, reducing protein synthesis. (Right) The monoclonal antibody evinacumab binds to and neutralizes circulating ANGPTL3 protein. Both strategies alleviate ANGPTL3-mediated inhibition of LPL. This enhances the hydrolysis of TGs in VLDL and IDL, promoting their clearance via hepatic receptors. Source: Created with BioRender.com

Inhibition of ANGPTL3, a regulator of LPL and endothelial lipase (EL) activity, reduces atherogenic lipoproteins and offers a novel mechanism for managing complex dyslipidemias. Figure 4 illustrates two therapeutic strategies targeting ANGPTL3.

##### Evinacumab

Evinacumab is a recombinant human monoclonal antibody specifically designed to bind circulating ANGPTL3. It is produced via cell culture using genetically engineered Chinese hamster ovary cells [67]. Evinacumab has undergone extensive evaluation through phase I–III clinical trials, spanning healthy volunteers to patients with homozygous familial hypercholesterolemia. A meta-analysis of five RCTs by Jin et al. reported that evinacumab reduced TG levels by approximately 51% without significant safety concerns [68]. In 2021, the Polish lipid association recommended evinacumab as adjunctive therapy for familial hypercholesterolemia management [97]. Furthermore, the 2019 ESC/EAS guidelines classified evinacumab as a novel approach for reducing TRLs and their remnants [89]. Emerging evidence also suggests that evinacumab may promote atherosclerotic plaque regression [98], highlighting its potential as an important agent for mitigating TG-associated residual ASCVD risk.

##### Vupanorsen (GalNAc-ASO)

The clinical development of vupanorsen was discontinued following phase III trials that demonstrated only modest lipid-lowering efficacy alongside concerning safety signals, including elevations in liver enzymes and hepatic fat accumulation [99].

The GalNAc-conjugated siRNA and ASO therapies primarily target hepatic cells and exhibit minimal renal exposure, thereby substantially reducing the risk of nephrotoxicity. However, the long-term safety profile of these agents beyond phase II/III clinical trials remains uncertain, particularly in patients with advanced CKD, who have been underrepresented in these studies. Furthermore, vigilant monitoring for potential off-target effects and immune activation is essential. Critically, definitive evidence regarding their efficacy in lowering cardiovascular and renal risks awaits the completion of ongoing cardiovascular outcome trials [65]. Given the considerable cost associated with these biologic therapies, it is imperative to identify patient subgroups most likely to achieve significant absolute benefit, such as individuals with severe, refractory HTG at high risk of pancreatitis or those with genetically confirmed dyslipidemias unresponsive to conventional treatments, to optimize cost-effectiveness and clinical utility.

### Ancillary therapies: agents with moderate TG-Lowering effects

#### Statins

Statins reduce TG levels by approximately 10–20%, primarily through inhibition of TRL synthesis and enhancement of receptor-mediated lipid clearance (Fig. 1) [70]. While their cardiovascular benefits are well established in the general population and patients with early-stage CKD [100], the efficacy of statins diminishes markedly in advanced CKD, especially among individuals undergoing dialysis [101, 102]. Large RCTs, including the 4D study [71] and the AURORA trial [72], failed to demonstrate significant cardiovascular outcome improvements with statin therapy in hemodialysis patients. This limited benefit is largely attributed to the unique cardiovascular pathophysiology in end-stage renal disease, where events such as sudden cardiac death and HF frequently arise from non-atherosclerotic mechanisms. Moreover, statin treatment has been associated with elevations in lipoprotein(a) levels, which may partially counterbalance their protective cardiovascular effects. Accordingly, the KDIGO Clinical Practice Guideline for Lipid Management in CKD generally advises against initiating statin therapy in patients who have already commenced dialysis [103].

#### Proprotein convertase Subtilisin/Kexin type 9 (PCSK9) inhibitors

**Fig. 5:**
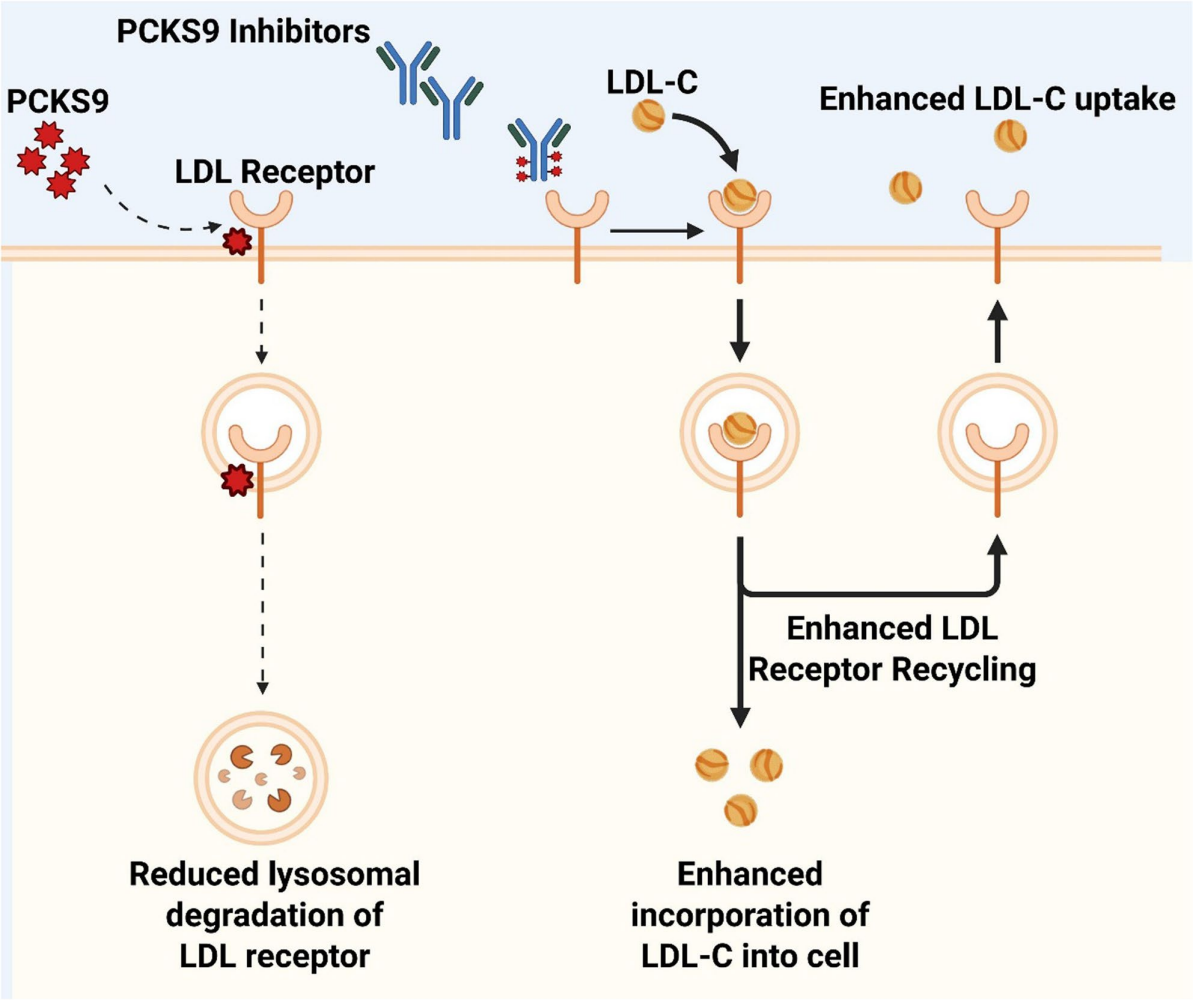
Mechanism of PCSK9 inhibitors. PCSK9 inhibitors neutralize circulating PCSK9, preventing its binding to hepatic LDL receptors. This reduces receptor degradation and promotes receptor recycling to the cell surface, thereby enhancing LDL-C uptake and clearance. Source: Created with BioRender.com

PCSK9 inhibitors exert their lipid-lowering effects by binding to and neutralizing circulating PCSK9, thereby preventing PCSK9-mediated degradation of hepatic LDL receptors and increasing receptor availability on the hepatocyte surface. This mechanism markedly enhances the hepatic clearance of LDL-C as well as very-low-density lipoprotein (VLDL) remnant particles (Fig. 5) [73]. Clinically, PCSK9 inhibitors produce profound reductions in LDL-C and are also associated with modest TG lowering, typically in the range of 15–25% [74]. Their cardiovascular efficacy has been firmly established in large-scale outcome trials, including the FOURIER and ODYSSEY programs [75, 76]. Importantly, subgroup analyses have demonstrated consistent lipid-lowering efficacy and favorable safety profiles across all stages of CKD [104, 105]. Consequently, in the dyslipidemia management of patients with CKD, PCSK9 inhibitors primarily serve as potent agents for intensive LDL-C reduction in individuals at very high risk of ASCVD. In patients with concomitant HTG, their ancillary TG-lowering effects further support their role as an effective therapeutic option for the treatment of severe mixed dyslipidemia.

#### Bempedoic acid

Bempedoic acid is an orally administered inhibitor of ATP-citrate lyase that produces a modest reduction in TG concentrations, typically in the range of 10–20% [77, 78]. Notably, its active metabolite is generated selectively within hepatocytes, which markedly limits systemic exposure and translates into a low risk of muscle-related adverse events [106]. In addition, because neither bempedoic acid nor its metabolites undergo renal excretion, dose adjustment is unnecessary across the full spectrum of CKD stages [79]. Evidence from the CLEAR Outcomes trial further demonstrated both cardiovascular benefit and a favorable safety profile in patients with CKD [79]. Collectively, these characteristics position bempedoic acid as a particularly suitable therapeutic alternative for statin-intolerant individuals with CKD, providing clinically meaningful lipid-lowering efficacy without compromising renal safety.

#### SGLT2i and GLP-1 RAs

SGLT2i and GLP-1 RAs have emerged as foundational therapies in the contemporary management of CKD, providing robust cardiorenal protection through mechanisms that extend beyond glucose lowering [80, 107]. Both drug classes are also associated with modest but clinically meaningful reductions in TG levels (approximately 10–15% with SGLT2i and 10–20% with GLP-1 RAs), thereby offering additional metabolic benefits to patients with concomitant HTG [81]. Their efficacy in slowing CKD progression and reducing hard cardiorenal outcomes has been consistently demonstrated in large cardiovascular outcome trials and dedicated renal studies, including CREDENCE, DAPA-CKD, and FLOW [82, 83, 85, 86]. Accordingly, in patients with CKD and HTG, irrespective of diabetes status, these agents should be prioritized early in the treatment algorithm to secure core cardiorenal benefits.

## Unresolved questions and conceptual advances

HTG in the context of CKD should not be regarded as a simple lipid abnormality, but rather as a central metabolic driver of the proposed “lipotoxicity–inflammation–fibrosis” cycle that accelerates both renal deterioration and cardiovascular risk. Despite this conceptual advance, several critical questions remain unanswered with respect to its translation into clinical practice.

### Defining lipotoxic phenotypes in CKD

Can lipid metabolic signatures and inflammatory response patterns of specific renal cell populations be accurately delineated across different stages of CKD? Is there a quantifiable and clinically actionable “lipotoxicity threshold” beyond which targeted intervention becomes necessary? Moreover, can distinct lipid species or circulating biomarkers be identified as early indicators or therapeutic targets of lipid-driven renal injury?

### Targeting the entire pathogenic cycle versus individual nodes

Do therapeutic strategies that simultaneously modulate multiple components of the “lipotoxicity–inflammation–fibrosis” axis yield synergistic benefits that exceed those achieved with sequential or single-agent interventions in preventing CKD progression?

### Long-term safety–efficacy balance in advanced CKD

What are the true long-term renal and systemic safety profiles of potent emerging therapies, such as GalNAc-conjugated RNA-based agents, in patients with stage 4–5 CKD or those receiving dialysis, populations that remain underrepresented in existing trials? In these high-risk groups, can profound TG reduction be translated into meaningful reductions in end-stage kidney disease incidence or specific cardiovascular events?

## Emerging research directions and future prospects

To address the key challenges outlined above and to rigorously validate the proposed management framework, future investigations should adopt advanced, integrative methodological approaches and undertake deeper mechanistic studies across targeted biological domains.

### Clarifying lipotoxicity phenotypes

The integration of single-cell and spatial transcriptomic technologies with imaging mass spectrometry enables high-resolution mapping of lipidomic signatures and inflammatory responses within discrete renal cell populations. This multimodal approach facilitates detailed characterization of lipid metabolic programs in podocytes, renal tubular epithelial cells, and infiltrating immune cells across different stages of CKD [108]. In parallel, human kidney organoids derived from individuals harboring specific genetic variants, such as those affecting ApoC-III or ANGPTL3, or experimentally exposed to hypertriglyceridemic conditions provide dynamic, patient-relevant platforms to define critical “lipotoxicity thresholds” and to evaluate targeted therapeutic interventions. Ultimately, the integration of these high-dimensional datasets using artificial intelligence–based analytical frameworks may enable the construction of predictive models that classify distinct renal “lipotoxicity phenotypes,” thereby supporting precision medicine approaches in CKD.

### Targeting novel molecular pathways

**Fig. 6:**
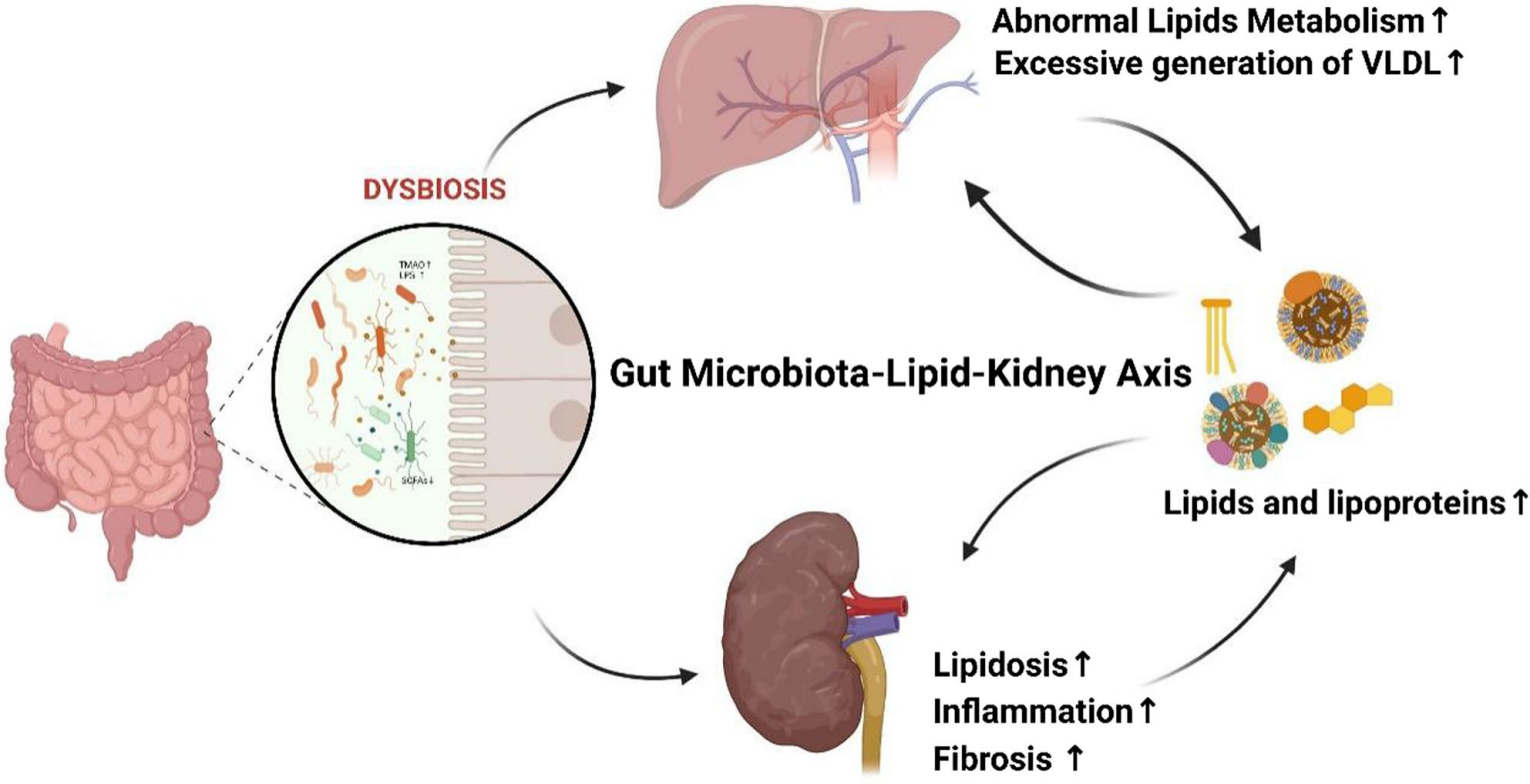
The role of the gut microbiota-lipid-kidney axis in the progression of CKD. Dysbiosis of the gut microbiota can lead to an increase in harmful metabolites (such as TMAO, LPS) and a decrease in beneficial metabolites (such as short-chain fatty acids), thereby promoting systemic inflammation, abnormal lipid metabolism, and increased production of VLDL. These factors collectively contribute to lipid deposition, inflammatory response, and renal fibrosis, forming a vicious cycle. As kidney function deteriorates, the accumulated uremic toxins (such as indoxyl sulfate) can further impair intestinal barrier function, exacerbating microbiota dysbiosis.Source: Created with BioRender.com

Beyond established mechanisms, several emerging molecular targets have shown promise for modulating renal lipid metabolism and attenuating de novo lipogenesis. In particular, acyl-CoA synthetase short-chain family member 2 and glucagon receptor signaling pathways have demonstrated the capacity to suppress lipid synthesis and improve renal lipid handling in preclinical studies [109–111]. In parallel, growing evidence highlights the gut microbiota–lipid–kidney axis (Fig. 6) as a tractable therapeutic domain. Future investigations should aim to delineate specific microbial taxa or functional communities whose metabolites, such as trimethylamine N-oxide or short-chain fatty acids, directly influence renal LPL activity and lipotoxic stress. Translational efforts should further evaluate innovative interventions, including next-generation prebiotics and probiotics as well as targeted inhibition of microbial metabolic enzymes, to determine their efficacy in modifying lipid-driven kidney injury [112, 113].

### Generating long-term evidence for novel therapies

For emerging RNA-based therapeutics and other precision-targeted interventions, the establishment of robust long-term evidence in advanced CKD necessitates the conduct of dedicated phase III and IV clinical trials that deliberately enroll adequate numbers of patients with moderate-to-advanced CKD, including those receiving dialysis. Such trials should adopt biomarker-guided enrichment strategies, such as preferential inclusion of individuals with elevated baseline ApoC-III levels, to enhance the identification of treatment-responsive subgroups. In parallel with the evaluation of efficacy and safety outcomes, these studies should prospectively integrate formal health economic and cost-effectiveness analyses to ensure that therapeutic benefits can be translated into sustainable, real-world clinical practice.

## Strengths and limitations

This review provides a timely and comprehensive synthesis of the complex bidirectional relationship between HTG and CKD. It proposes an integrated pathophysiological framework centered on a “lipotoxicity–inflammation–fibrosis” cycle, thereby linking disturbances in lipid metabolism to both CKD progression and increased cardiovascular risk. Furthermore, by critically appraising evidence related to renal safety and cardiorenal outcome benefits, the review develops a pragmatic, phenotype-based management framework tailored to this patient population. Nevertheless, as a narrative review, the analysis is inherently shaped by the authors’ selection and interpretation of the available literature, introducing the potential for selection bias. Although considerable effort was made to incorporate recent and influential studies, the field, particularly with respect to emerging therapeutic modalities, is evolving rapidly, and some of the most recent clinical trial data or updated guideline recommendations may not yet be included. In addition, while the proposed stratified treatment strategies offer meaningful clinical insights, the absence of a formal cost-effectiveness evaluation of RNA-based therapies across different CKD subpopulations represents a notable limitation, given that economic feasibility is a critical determinant of real-world clinical implementation.

## Conclusion

In the management of HTG among patients with CKD, therapeutic strategies should evolve beyond conventional lipid-lowering approaches toward a comprehensive and integrated framework that addresses interconnected pathogenic pathways, including lipotoxicity, inflammation, fibrosis, and heightened cardiovascular risk. SGLT2i and GLP-1 RAs, as foundational therapies, exert favorable metabolic effects while also demonstrating anti-inflammatory and anti-fibrotic properties, thereby conferring broad cardio-renal protection in CKD patients with HTG. In individuals who continue to exhibit significant HTG and residual cardiovascular risk despite optimized cornerstone therapy, adjunctive treatment with IPE or pemafibrate, agents with more favorable safety profiles in advanced CKD, may be considered to directly attenuate circulating lipotoxic burden. For patients presenting with severe mixed dyslipidemia or intolerance to statins, PCSK9 inhibitors or bempedoic acid represent alternative options for mitigating concomitant atherosclerotic cardiovascular risk. In cases of severe and refractory HTG attributable to specific genetic abnormalities, emerging RNA-based therapies provide targeted upstream interventions that reduce the production of lipotoxic substrates and have demonstrated considerable therapeutic potential. Overall, the implementation of stratified, stage-specific management strategies for HTG across the spectrum of CKD may facilitate truly personalized, mechanism-driven treatment, ultimately advancing the overarching objective of improving hard cardiorenal clinical outcomes.

## Data Availability

All studies reviewed by the authors for this manuscript are accessible through PubMed, with digital object identifiers listed in the references to facilitate retrieval and access.

## Abbreviations

ANGPTL: Angiopoietin-like Protein
ApoC-III: Apolipoprotein C-III
CHD: Coronary Heart Disease
CKD: Chronic Kidney Disease
CVD: Cardiovascular disease
eGFR: Estimated Glomerular Filtration Rate
FCS: Familial Chylomicronemia Syndrome
HDL-C: High-Density Lipoprotein Cholesterol
HF: Heart Failure
HTG: Hypertriglyceridemia
GLP-1: Glucagon-like peptide-1
LDL-C: Low-Density Lipoprotein Cholesterol
LPL: Lipoprotein Lipase
PPARα: Peroxisome proliferator–activated receptor alpha
RNAi: RNA interference
ROS: reactive oxygen species
SGLT-2: Sodium-glucose co-transporter 2
TG: Triglyceride
TRL: Triglyceride-rich Lipoprotein
VLDL: Very Low-Density Lipoproteins
